# Postoperative Functional Recovery After Carpal Tunnel Release: A Narrative Review on Exercise-Based Rehabilitation

**DOI:** 10.7759/cureus.103195

**Published:** 2026-02-08

**Authors:** Evangelos E Tzanis, Sophia Syngouna, Evangelos Sakellariou, John Vlamis

**Affiliations:** 1 3rd Orthopaedic Department, National and Kapodistrian University of Athens, KAT General Hospital, Athens, GRC; 2 Hand-Upper Limb and Microsurgery Department, KAT General Hospital of Attica, Kifisia, GRC

**Keywords:** carpal tunnel release, carpal tunnel syndrome, exercise-based rehabilitation, grip strength, postoperative rehabilitation

## Abstract

Carpal tunnel release is a commonly performed surgical procedure that effectively alleviates compressive symptoms in patients with carpal tunnel syndrome. However, postoperative recovery of hand function, including grip strength, pinch strength, and coordinated hand use, varies considerably among individuals. This variability indicates that functional recovery is influenced by factors beyond the surgical intervention itself. This narrative review synthesizes the existing literature on postoperative functional recovery following carpal tunnel release, with emphasis on functional outcomes, rehabilitation strategies, and exercise-based interventions. Surgical decompression consistently improves sensory symptoms; however, restoration of hand strength and overall functional performance is frequently delayed and heterogeneous. Although early mobilization is generally considered safe, it does not adequately address persistent deficits in strength, coordination, or endurance. Exercise-based rehabilitation approaches-including nerve and tendon gliding exercises and progressive strengthening-are biologically plausible and potentially relevant to postoperative recovery, yet the existing literature is characterized by variability in study design, intervention protocols, and outcome assessment. Postoperative recovery following carpal tunnel release represents a multifactorial process. Exercise-based rehabilitation is a promising but insufficiently studied modifiable component of postoperative care. Well-designed prospective studies are required to clarify its role in optimizing functional recovery after carpal tunnel surgery.

## Introduction and background

Carpal tunnel syndrome (CTS) is the most common compressive neuropathy of the upper extremity and represents a frequent cause of pain, sensory disturbance, and functional impairment in the general population [1-5]. The condition is associated with substantial personal, occupational, and socioeconomic burden, particularly due to its impact on hand function and activities of daily living [1,2]. When conservative management fails or when neurological compromise progresses, surgical decompression of the transverse carpal ligament is widely accepted as the definitive treatment [6,7]. Carpal tunnel release (CTR) is a well-established procedure and has consistently demonstrated high rates of symptom relief and patient satisfaction. Sensory symptoms such as pain, numbness, and paresthesia often improve relatively quickly following decompression; however, postoperative recovery of hand function is not uniform across individuals. Although surgical success is commonly defined by symptomatic improvement, restoration of grip strength, pinch strength, and coordinated hand use may be delayed or incomplete in a subset of patients [8-12]. Several studies have shown that improvements in motor performance frequently lag behind sensory recovery, highlighting a dissociation between symptom resolution and functional restoration [8-11]. This variability suggests that postoperative outcomes are influenced by factors beyond the technical aspects of surgery alone. Postoperative functional recovery following CTR is increasingly recognized as a multifactorial process involving neuromuscular adaptation, musculoskeletal conditioning, and behavioral factors such as protective movement patterns and temporary disuse [9-12]. Objective measures of hand strength, particularly grip and pinch strength, are therefore commonly used to assess recovery, as they provide reproducible and clinically meaningful indicators of functional performance [12]. Understanding the determinants of postoperative functional recovery is clinically relevant, particularly given the variability in postoperative management following carpal tunnel release. While early mobilization is generally considered safe, its role in optimizing functional outcomes remains uncertain, and the contribution of structured exercise-based rehabilitation has not been clearly defined. The purpose of this review is to examine postoperative functional recovery following carpal tunnel release, with particular emphasis on objective strength outcomes and the potential role of exercise-based rehabilitation, in order to inform clinical decision-making and future research.

## Review

Postoperative functional recovery after carpal tunnel release

Carpal tunnel release is associated with predictable improvement in pain and paresthesia; however, functional recovery of the hand frequently follows a more variable and protracted course [6-12]. Although decompression effectively addresses median nerve compression, postoperative recovery should not be regarded as a uniform or purely time-dependent process. Multiple studies have reported that improvements in grip strength and pinch strength often lag behind sensory recovery, with some patients experiencing transient postoperative weakness or delayed functional gains despite technically successful decompression [8-11]. This dissociation between symptom relief and restoration of hand function underscores the complex and multifactorial nature of postoperative recovery. Functional recovery following carpal tunnel release encompasses not only neural recovery but also neuromuscular, behavioral, and musculoskeletal components. Objective assessment of recovery commonly relies on measurements of grip and pinch strength owing to their reproducibility and relevance to daily, occupational, and recreational activities [12,13]. Reported timelines for strength recovery vary substantially across studies, even among cohorts undergoing similar surgical techniques. Factors such as postoperative pain, protective movement behavior, transient disuse, altered motor recruitment patterns, and the duration and severity of preoperative symptoms have been proposed to contribute to this variability [9-12,14]. Historically, postoperative wrist immobilization and splinting were routinely incorporated intopostoperative management following carpal tunnel release, based on the assumption that limiting motion would protect the surgical site and facilitate recovery. Subsequent prospective and comparative investigations, however, have failed to demonstrate consistent functional benefits of prolonged immobilization when compared with early mobilization strategies [15,16]. In particular, prospective evidence indicates that routine wrist immobilization does not confer additional functional advantages and does not appear to favorably influence the trajectory of postoperative recovery, thereby questioning the clinical value of predominantly passive postoperative strategies [15-17].

Grip and pinch strength as functional outcomes

Grip and pinch strength are widely used objective indicators of functional recovery following carpal tunnel release, reflecting the capacity of the hand to perform both power and precision tasks after median nerve decompression [8-13]. While surgical decompression effectively alleviates pain and paresthesia in the majority of patients, functional outcomes-particularly those related to strength, endurance, and dexterity-demonstrate greater inter-individual variability [6-10]. Dynamometric assessment of grip strength is frequently employed in both clinical and research settings due to its practicality, reproducibility, and established reliability and validity, with normative reference values enabling comparisons across populations and time points [13]. Pinch strength measurements provide complementary information on fine motor performance and intrinsic muscle function, which may be differentially affected during the postoperative recovery process [9,11,12]. Several investigations have described a characteristic temporal pattern of postoperative strength recovery, whereby grip strength may initially decline during the early postoperative period before gradually improving over subsequent follow-up intervals [8-11]. Temporal analyses further suggest that grip and pinch strength do not necessarily recover in parallel and may follow distinct recovery trajectories after open carpal tunnel release, with considerable inter-individual variability in both the magnitude and timing of functional restoration [11]. These observations support the concept that postoperative recovery following carpal tunnel release is multifactorial and not solely determined by the technical success of surgical decompression [6,7,12]. The transient reduction in postoperative force output has been attributed to a combination of factors, including postoperative pain, protective movement behavior, short-term disuse, and altered motor recruitment patterns, rather than persistent neural impairment [9-12]. Alternative indicators of motor recovery, such as manual muscle testing and assessment of thenar muscle atrophy, have also been proposed; however, systematic evaluation suggests that these measures demonstrate lower sensitivity to change compared with dynamometric assessments and may fail to capture subtle yet clinically meaningful functional improvements over time [12]. Collectively, the available evidence indicates that grip- and pinch-strength outcomes reflect a complex interaction between neural recovery, musculoskeletal adaptation, and behavioral factors, underscoring their potential responsiveness to targeted postoperative rehabilitation strategies [14-16]. Recent evidence syntheses have not supported routine postoperative splinting as a strategy that consistently improves functional outcomes after carpal tunnel release [17].

Exercise-based rehabilitation after carpal tunnel release

Exercise-based rehabilitation has been proposed as a potential contributor to postoperative recovery following carpal tunnel release, particularly in the context of persistent deficits in strength, coordination, and endurance [18-21]. Therapeutic exercise is frequently introduced alongside early mobilization protocols with the aim of facilitating neuromuscular adaptation, restoring coordinated motor patterns, and supporting the return to functional hand use [15,17]. Early clinical research has suggested that structured exercise interventions may positively influence symptom severity and selected functional outcomes in individuals with carpal tunnel syndrome, providing a conceptual foundation for postoperative application [21]. These observations support the hypothesis that active rehabilitation strategies may address neuromuscular impairments that are not fully resolved through surgical decompression alone. Nerve and tendon gliding exercises are among the most commonly described rehabilitation modalities. These exercises are designed to promote relative motion between neural structures and surrounding soft tissues within the carpal tunnel, with proposed mechanisms including facilitation of tendon excursion, reduction of perineural adhesion formation, and optimization of intraneural circulation [18-20]. Clinical studies evaluating nerve and tendon gliding exercises have reported improvements in selected clinical and functional outcomes, although the magnitude and consistency of these effects vary across studies [18-21]. Progressive strengthening exercises represent an additional component of postoperative rehabilitation, targeting muscle groups affected by preoperative compression, postoperative disuse, or altered motor recruitment patterns. Given that postoperative force production is influenced by muscle conditioning, coordination, and motor control in addition to neural recovery, strengthening interventions may be particularly relevant for functional restoration [13,14]. Nevertheless, substantial heterogeneity in exercise prescription across studies, including variation in initiation time, intensity, frequency, duration, and supervision, has been consistently reported [14,18-21], limiting definitive conclusions regarding efficacy.

For clarity, we summarize the key themes of the recent evidence below in a structured format. Structured summary of recent evidence (text-based overview): Across the available postoperative literature, exercise-based approaches most commonly include (i) nerve and tendon gliding exercises, and (ii) progressive strengthening programs initiated after the early postoperative phase. Outcomes are typically evaluated using objective strength measures (grip and pinch) and patient-reported functional assessments, with follow-up timing varying substantially between studies. Overall, the evidence suggests that early mobilization alone is generally safe but may be insufficient to address persistent deficits in strength, coordination, or endurance, whereas exercise-based rehabilitation is biologically plausible and may support functional recovery; however, conclusions remain limited by heterogeneity in intervention timing, intensity, supervision, and outcome assessment across studies.

Clinical and research implications

From a clinical perspective, the findings summarized in this review highlight the importance of distinguishing symptomatic improvement from functional recovery following carpal tunnel release. While surgical decompression reliably alleviates pain and paresthesia, residual deficits in strength and coordination may persist and influence return to work and activities of daily living [6-12]. Objective strength assessments may therefore provide valuable complementary information when evaluating postoperative outcomes and guiding rehabilitation decisions [12-14]. From a research standpoint, the heterogeneity observed across exercise-based interventions underscores the need for standardized rehabilitation protocols, clearly defined progression criteria, and objective functional endpoints [14,18-21]. Such considerations are essential for advancing the evidence base and informing future prospective studies examining the role of therapeutic exercise in postoperative recovery following carpal tunnel release.

Discussion and future directions

The evidence synthesized in this review indicates that postoperative recovery following carpal tunnel release extends beyond relief of compressive symptoms and involves a multifactorial process of functional reintegration of the hand [6-12]. While surgical decompression effectively addresses median nerve compression, restoration of strength and coordinated hand use frequently follows a delayed and heterogeneous course [6-11]. Evidence syntheses and systematic reviews indicate that early mobilization is safe and appropriate but does not directly address persistent functional deficits [15-17]. Long-term outcome analyses further suggest that residual functional limitations may persist despite satisfactory symptomatic improvement, emphasizing the potential influence of postoperative rehabilitation strategies on long-term recovery trajectories [22]. Within this context, exercise-based rehabilitation emerges as a biologically plausible and potentially modifiable component of postoperative care. From a mechanistic perspective, experimental and physiological studies demonstrate that muscle function and force production are influenced by neuromuscular coordination, loading patterns, and training stimuli, independent of neural decompression alone [23]. Although such findings are not specific to carpal tunnel syndrome, they provide a theoretical framework supporting the incorporation of progressive strengthening into postoperative rehabilitation protocols. Despite this rationale, the existing literature is characterized by heterogeneity in intervention protocols, limited supervision, and inconsistent outcome assessment, which preclude definitive conclusions regarding efficacy [14,18-21]. Future prospective investigations employing structured, standardized exercise programs with clearly defined progression criteria and objective functional endpoints are warranted. Adequate sample sizes, standardized assessment timelines, and rigorous methodological design will be essential to clarify the role of therapeutic exercise in optimizing postoperative recovery after carpal tunnel release.

## Conclusions

Carpal tunnel release is effective in alleviating sensory symptoms associated with carpal tunnel syndrome; however, functional recovery of the hand is neither uniform nor automatic. Restoration of strength and coordinated hand use frequently lags behind symptom resolution, underscoring the multifactorial nature of postoperative recovery. Exercise-based rehabilitation represents a biologically plausible and potentially modifiable determinant of functional outcome, yet the current body of evidence is limited by methodological heterogeneity across studies. Well-designed prospective investigations are required to clarify the role of structured exercise programs in optimizing functional recovery after carpal tunnel surgery.

## References

[1] Ibrahim I, Khan WS, Goddard N, Smitham P (2012). Carpal tunnel syndrome: a review of the recent literature. Open Orthop J.

[2] Lewis C, Mauffrey C, Newman S, Lambert A, Hull P (2010). Current concepts in carpal tunnel syndrome. Eur J Orthop Surg Traumatol.

[3] Urits I, Gress K, Charipova K, Orhurhu V, Kaye AD, Viswanath O (2019). Recent advances in the understanding and management of carpal tunnel syndrome: a comprehensive review. Curr Pain Headache Rep.

[4] Werner RA, Andary M (2002). Carpal tunnel syndrome: pathophysiology and clinical neurophysiology. Clin Neurophysiol.

[5] Padua L, Coraci D, Erra C (2016). Carpal tunnel syndrome: clinical features, diagnosis, and management. Lancet Neurol.

[6] Badger SA, O'Donnell ME, Sherigar JM, Connolly P, Spence RA (2008). Open carpal tunnel release--still a safe and effective operation. Ulster Med J.

[7] Murthy PG, Goljan P, Mendez G, Jacoby SM, Shin EK, Osterman AL (2015). Mini-open versus extended open release for severe carpal tunnel syndrome. Hand (N Y).

[8] Young VL, Logan SE, Fernando B, Grasse P, Seaton M, Young AE (1992). Grip strength before and after carpal tunnel decompression. South Med J.

[9] Gellman H, Kan D, Gee V, Kuschner SH, Botte MJ (1989). Analysis of pinch and grip strength after carpal tunnel release. J Hand Surg Am.

[10] Olsen KM, Knudson DV (2001). Change in strength and dexterity after open carpal tunnel release. Int J Sports Med.

[11] Netscher D, Steadman AK, Thornby J, Cohen V (1998). Temporal changes in grip and pinch strength after open carpal tunnel release and the effect of ligament reconstruction. J Hand Surg Am.

[12] Geere J, Chester R, Kale S, Jerosch-Herold C (2007). Power grip, pinch grip, manual muscle testing or thenar atrophy - which should be assessed as a motor outcome after carpal tunnel decompression? A systematic review. BMC Musculoskelet Disord.

[13] Mathiowetz V, Weber K, Volland G, Kashman N (1984). Reliability and validity of grip and pinch strength evaluations. J Hand Surg Am.

[14] Multanen J, Uimonen MM, Repo JP, Häkkinen A, Ylinen J (2021). Use of conservative therapy before and after surgery for carpal tunnel syndrome. BMC Musculoskelet Disord.

[15] Cook AC, Szabo RM, Birkholz SW, King EF (1995). Early mobilization following carpal tunnel release. A prospective randomized study. J Hand Surg Br.

[16] Martins RS, Siqueira MG, Simplício H (2006). Wrist immobilization after carpal tunnel release: a prospective study. Arq Neuropsiquiatr.

[17] Peter-Okaka UI, Shiri S, Owodunni O (2024). Are there any benefits for post-operative splinting after carpal tunnel release? A systematic review and meta-analysis. BMC Musculoskelet Disord.

[18] Rozmaryn LM, Dovelle S, Rothman ER, Gorman K, Olvey KM, Bartko JJ (1998). Nerve and tendon gliding exercises and the conservative management of carpal tunnel syndrome. J Hand Ther.

[19] Akalin E, El O, Peker O (2002). Treatment of carpal tunnel syndrome with nerve and tendon gliding exercises. Am J Phys Med Rehabil.

[20] Horng YS, Hsieh SF, Tu YK, Lin MC, Horng YS, Wang JD (2011). The comparative effectiveness of tendon and nerve gliding exercises in patients with carpal tunnel syndrome: a randomized trial. Am J Phys Med Rehabil.

[21] Thomas RE, Butterfield RK, Hool JN, Herrick RT (1993). Effects of exercise on carpal tunnel syndrome symptoms. Appl Ergon.

[22] Louie D, Earp B, Blazar P (2012). Long-term outcomes of carpal tunnel release: a critical review of the literature. Hand (N Y).

[23] Leger AB, Milner TE (2001). Muscle function at the wrist after eccentric exercise. Med Sci Sports Exerc.

